# From the Farms to the Dining Table: The Distribution and Molecular Characteristics of Antibiotic-Resistant *Enterococcus* spp. in Intensive Pig Farming in South Africa

**DOI:** 10.3390/microorganisms9050882

**Published:** 2021-04-21

**Authors:** Sasha Badul, Akebe L. K. Abia, Daniel G. Amoako, Keith Perrett, Linda A. Bester, Sabiha Y. Essack

**Affiliations:** 1 Antimicrobial Research Unit, College of Health Sciences, University of KwaZulu-Natal, Durban 4000, South Africa; sashabadul@yahoo.co.uk (S.B.); lutherkinga@yahoo.fr (A.L.K.A.); amoakodg@gmail.com (D.G.A.); 2 Biomedical Resource Unit, College of Health Sciences, University of KwaZulu-Natal, Durban 4000, South Africa; besterl@ukzn.ac.za; 3 Centre for Respiratory Diseases and Meningitis, National Institute for Communicable Diseases, Johannesburg 2131, South Africa; 4 Epidemiology Section, KwaZulu-Natal Agriculture & Rural Development-Veterinary Service, Pietermaritzburg 3201, South Africa; keith.perrett@kzndard.gov.za

**Keywords:** antibiotic resistance, *Enterococcus* spp., multidrug resistance, farm-to-fork, intensive pig farming, public health, biosecurity, food safety, virulence genes, one health

## Abstract

Foodborne pathogens, including antibiotic-resistant species, constitute a severe menace to food safety globally, especially food animals. Identifying points of concern that need immediate mitigation measures to prevent these bacteria from reaching households requires a broad understanding of these pathogens’ spread along the food production chain. We investigated the distribution, antibiotic susceptibility, molecular characterization and clonality of *Enterococcus* spp. in an intensive pig production continuum in South Africa, using the farm-to-fork approach. *Enterococcus* spp. were isolated from 452 samples obtained along the pig farm-to-fork continuum (farm, transport, abattoir, and retail meat) using the IDEXX Enterolert^®^/Quanti-Tray^®^ 2000 system. Pure colonies were obtained on selective media and confirmed by real-time PCR, targeting genus- and species-specific genes. The susceptibility to antibiotics was determined by the Kirby–Bauer disk diffusion method against 16 antibiotics recommended by the WHO-AGISAR using EUCAST guidelines. Selected antibiotic resistance and virulence genes were detected by real-time PCR. Clonal relatedness between isolates across the continuum was evaluated by REP-PCR. A total of 284 isolates, consisting of 79.2% *E. faecalis*, 6.7% *E. faecium*, 2.5% *E. casseliflavus*, 0.4% *E. gallinarum*, and 11.2% other *Enterococcus* spp., were collected along the farm-to-fork continuum. The isolates were most resistant to sulfamethoxazole-trimethoprim (78.8%) and least resistant to levofloxacin (5.6%). No resistance was observed to vancomycin, teicoplanin, tigecycline and linezolid. *E. faecium* displayed 44.4% resistance to quinupristin-dalfopristin. Also, 78% of the isolates were multidrug-resistant. Phenotypic resistance to tetracycline, aminoglycosides, and macrolides was corroborated by the presence of the *tet*M, *aph(3′)-IIIa*, and *erm*B genes in 99.1%, 96.1%, and 88.3% of the isolates, respectively. The most detected virulence gene was *gel*E. Clonality revealed that *E*. *faecalis* isolates belonged to diverse clones along the continuum with major REP-types, mainly isolates from the same sampling source but different sampling rounds (on the farm). *E. faecium* isolates revealed a less diverse profile. The results suggest that intensive pig farming could serve as a reservoir of antibiotic-resistant bacteria that could be transmitted to occupationally exposed workers via direct contact with animals or consumers through animal products/food. This highlights the need for more robust guidelines for antibiotic use in intensive farming practices and the necessity of including *Enterococcus* spp. as an indicator in antibiotic resistance surveillance systems in food animals.

## 1. Introduction

Food safety is among the pressing human priorities due to the numerous foodborne disease outbreaks that kill hundreds of thousands of people around the world yearly [1]. These diseases are associated with the consumption of food contaminated by microbial agents (bacteria, viruses, fungi, parasites) or toxins produced by some of these organisms [2]. Even more concerning is that some of these organisms, especially bacteria, have developed resistance to most of the antibiotics initially used to kill them. Although antibiotic resistance (ABR) occurs naturally, its emergence has been exacerbated by the inappropriate and excessive use of antibiotics, poor therapy adherence, over-use of antibiotics in food-producing animals, and poor hygiene and sanitation [3]. A direct consequence of ABR is the failure to successfully treat infections, which leads to increased mortality, prolonged illness, and reduced livelihood and food security. With the rise in ABR and a decline in new antibiotic discovery and development, it is imperative to monitor the emergence and spread of ABR in humans and (food) animals.

Antibiotic-resistant bacteria can develop and move between food-producing animals and humans by direct exposure or through the food chain and the environment, irrespective of geographical or ecological borders [3]. The global rise in meat and meat products demands has led to a shift in farming practices, with a larger proportion of animals projected to be raised in cost-effective intensive farming systems [4]. In such farming systems, the high animal population densities and sub-optimal vaccination, sub-optimal biosecurity, and animal husbandry practices result in the over-reliance on antibiotics for the prophylactic and metaphylactic management of infections resulting in the emergence and spread of antibiotic-resistant bacterial species [5,6]. It is currently projected that the use of antibiotics in food animals will rise by 67% by 2030, two-thirds of which is expected to be used in intensive food animal production, with the use in pig and poultry production expected to double [7]. Therefore, it is crucial to understand how this could affect the spread of resistant bacteria through the food chain by studying the distribution of these species along entire food production chains.

Enterococci are Gram-positive bacteria and commensals in the gut of animals and humans, and as such, they provide information on the flow of Gram-positive resistance traits in the food chain [8,9]. They are opportunistic pathogens and serve as reservoirs of resistance genes that can be transferred to human pathogens transiting the intestinal tract [9,10]. *E. faecalis*, *E. faecium*, *E. hirae*, and *E. durans* are the most prevalent enterococcal species in the microbiota of humans and other mammals. *E. casseliflavus*, *E. gallinarum*, *E. avium*, and *E. cecorum* have also been reported in the microbiota of pigs, although in a lesser proportion [11]. *E. faecalis* and *E. faecium* are two of the most clinically important species [12]. The pathogenicity of *Enterococcus* spp. is enhanced by the expression of various virulence and antibiotic resistance genes that have been mobilized on diverse mobile genetic elements and are transferred by horizontal gene transfer (HGT).

There is currently limited data on the molecular epidemiology of antibiotic-resistant enterococci in pigs in South Africa, and no study has been conducted to investigate this along the farm-to-fork continuum. Obtaining such information could help the veterinary and public health sector develop strategies to prevent the spread of resistant bacteria across the food animal production system and their subsequent transmission to the dining table in households and other food outlets. This study, therefore, investigated the molecular epidemiology of antibiotic-resistant *Enterococcus* spp. from pigs in a food production continuum from farms to retail meat products in South Africa.

## 2. Materials and Methods

### 2.1. Ethical Clearance

Ethical approval was obtained from the Animal Research Ethics Committee (Reference: AREC/007/018; approved on 9 February 2018) and the Biomedical Research Ethics Committee (Reference: BCA444/16; approved on 17 March 2019) of the University of KwaZulu-Natal. This study also received permission under the terms of Section 20A of the Animal Diseases Act, 1984 (Act no. 35 of 1984) from The South African National Department of Agriculture, Forestry and Fisheries (Reference: 12/11/1/5; approved on 1 September 2018).

### 2.2. Study Population and Sampling Strategy

The study was conducted over four months (September 2018–January 2019) at an intensive pig farm and its associated abattoir in the uMgungundlovu District of KwaZulu-Natal, South Africa, as recommended by the WHO-AGISAR (World Health Organization on Integrated Surveillance of Antimicrobial Resistance (WHO-AGISAR) protocol guidelines [9] and processed as previously described [13]. Briefly, samples were collected following the farm-to-fork approach: from the growth period on the farm (fresh pig feces and wastewater/slurry), transport (truck swabs), abattoir (carcass swabs, carcass rinsate, and caecal contents), and retail meat (body, head, and thigh). Farm samples were collected bi-monthly over 18 weeks. samples were transported on ice to the laboratory and processed within 6 h after collection.

### 2.3. Isolation and Purification of Enterococcus spp.

The collected samples were diluted 1:10 in sterilized distilled water and vortexed. The samples were then processed using the Enterolert^®^ (IDEXX Laboratories, Inc., Westbrook, ME, USA) defined substrate according to the manufacturer. Briefly, following the samples’ dilution, appropriate volumes (10–100 µL) were topped up to 100 mL with sterile deionized water. After adding the Enterolert reagent, samples were transferred to Quanti-Trays, sealed, and incubated for 24 h at 41 °C. After 24 h, cells were harvested from fluorescent wells (observed under UV light) as previously described [14], streaked onto Chromocult^®^ enterococci agar (Merck, Darmstadt, Germany), and incubated for 24 h at 37 °C to obtained pure colonies. Red colonies presumptive of *Enterococcus* spp. were further purified by streaking onto bile-esculin agar (Lab M, Lancashire, UK) and incubating for 24 h at 37 °C. Single dark colonies were selected stored in 10% glycerol stock solutions at −80 °C for further analysis. *Enterococcus faecalis* ATCC 29212 was used as the control strain.

### 2.4. Molecular Confirmation and Speciation of Enterococcus spp.

Real-time PCR was used for the confirmation of *Enterococcus* and identification to the species level. DNA was extracted from stock cultures using the heat lysis method as previously described [15] after an initial resuscitation on nutrient agar. Extracted DNA (3 µL) was used as the template in a 10-µL reaction volume made up of 5 µL PowerUp™ SYBR™ Green master mix (ThermoFisher Scientific, Waltham, MA, USA), 0.5 µL of each forward and reverse primers (final concentration, 0.5 µM), and 1 µL of nuclease-free water. The positive controls, oligonucleotide primer pairs, thermal cycling conditions and melt-curve analysis were as previously described by Molechan et al. [15], with slight modifications on the initial activation in the cycling conditions for the detection of *Enterococcus* genus. Here, the initial activation consisted of an initial incubation at 50 °C for 2 min, followed by a second one at 95 °C for 2 min.

Similarly, the speciation of *E. faecalis*, *E. faecium*, *E. gallinarum*, and *E. casseliflavus*, was carried out in a total volume of 10 µL with the PowerUp™ SYBR™ Green replaced by the Luna^®^ Universal qPCR master mix (New England Biolabs, Ipswich, MA, USA). The positive controls, oligonucleotide primer pairs, thermal cycling conditions and melt-curve analysis were as previously described by Molechan et al. [15], with an increase in the number of cycles from 30 to 35.

All reactions were carried out in a QuantStudio™ 5 Real-Time PCR System (ThermoFisher Scientific, Waltham, MA, USA) included positive controls and No Template Controls (NTC) consisting of the PCR mix with template DNA replaced by nuclease-free water.

### 2.5. Antimicrobial Susceptibility Testing

The Kirby–Bauer disc diffusion method was used to ascertain antibiotic susceptibility against the antibiotic panel recommended by WHO-AGISAR [9] using the European Committee on Antimicrobial Susceptibility Testing [16] guidelines, except where breakpoints were absent, in which case the Clinical and Laboratory Standards Institute [17] ones were used. The antibiotics tested included ampicillin (10 µg), imipenem (10 µg), ciprofloxacin (15 µg), levofloxacin (15 µg), gentamicin (120 µg), streptomycin (300 µg), teicoplanin (30 µg), vancomycin (30 µg), quinupristin-dalfopristin (15 µg; *E. faecium* only), tigecycline (15 µg), linezolid (30 µg), nitrofurantoin (300 µg), sulfamethoxazole-trimethoprim (25 µg), erythromycin (15 µg), tetracycline (30 µg), and chloramphenicol (30 µg) (Oxoid, Basingstoke, UK).

### 2.6. Molecular Detection of Antibiotic Resistance and Virulence Genes

Selected genes encoding tetracycline (*tetK* and *tet*M), erythromycin (*erm*B), gentamycin (*aac(6′)-Ie-aph(2″)-Ia*), and streptomycin (*aph(3′)-IIIa*) resistance were investigated. Furthermore, genes encoding for virulence determinants in enterococci, including gelatinase production (*gel*E), sex pheromone (*cpd*), cytolysin (*cyl*A and *cyl*B) and cell wall adhesins (*efaAfs* and *efaAfm*), were also investigated. All genes were amplified in separate real-time PCR assays using specific primers and conditions previously described [15].

### 2.7. Clonality

The clonal distribution among selected MDR *E*. *faecalis* isolates (*n* = 99) and all *E. faecium* isolates (*n* = 19) were characterized with repetitive extragenic palindromic-PCR (REP-PCR) using the (GTG) 5 primer as described by [15]. Briefly, following DNA extraction with the GeneJET Genomic DNA purification kit (ThermoFisher Scientific, Waltham, MA, USA) based on the manufacturer’s manual, amplification was carried out in a 25-µL reaction volume. The products were separated by gel electrophoresis, visualized with a Gel Doc™ XR+ imaging system (Bio-Rad, Hercules, CA, USA), and the images were analyzed using the Bionumerics software version 6.6 (Applied Maths NV, Sint-Martens-Latem, Belgium) with clustering obtained at a ≥70.0% similarity cut-off value.

## 3. Results

### 3.1. Prevalence of Enterococcus spp.

**Figure 1:**
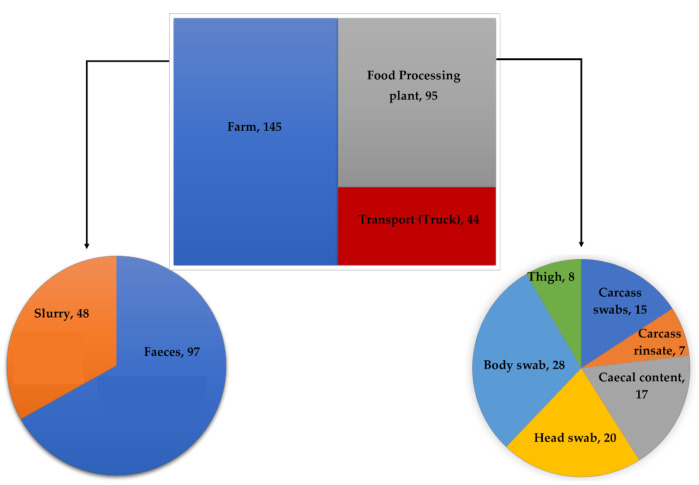
The overall distribution of *Enterococcus* spp. according to source across the farm-to-fork continuum.

Two hundred eighty-four (284) isolates were obtained along the farm-to-fork continuum of which, 145 (51.1%), 44 (15.5%), 39 (13.7%), and 56 (19.7%) were obtained from the farm, transport, abattoir, and retail sampling points, respectively (Figure 1).

**Figure 2:**
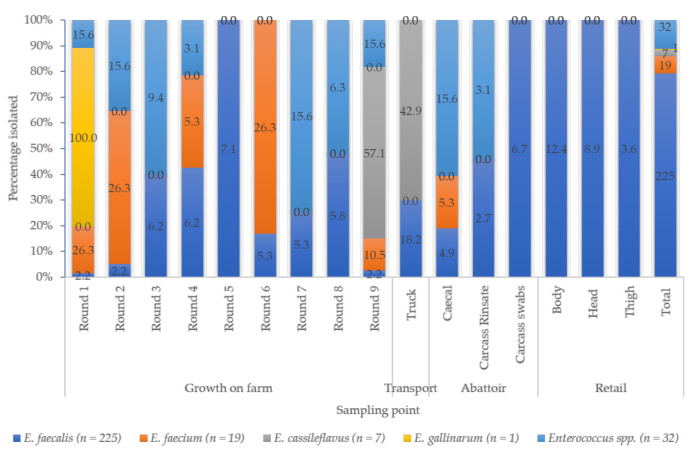
Distribution of various *Enterococcus* species along the farm-to-fork continuum.

Of the 284, 225 (79.2%) were *E. faecalis*, 19 (6.7%) were *E. faecium*, 7 (2.5%) were *E. casseliflavus*, 1 (0.4%) were *E. gallinarum*, while 32 (11.2%) were classified as “other *Enterococcus* spp.” (Figure 2)**.** *E. faecalis* was the most detected species throughout the continuum, while *E. faecium* was mainly isolated on the farm (Round 1, 2, and 6). No *E. faecium* isolates were recovered from transport or retail sampling points. There was a low prevalence of *E. casseliflavus* and *E. gallinarum*, with a few isolates being identified from the farm (Round 1 and 9) and transport vehicles (Figure 2).

### 3.2. Antibiotic Susceptibility Profile of Isolates

All the isolated tested in this study (100%) were susceptible to ampicillin, imipenem, teicoplanin, vancomycin, and linezolid. However, the isolates displayed a high percentage of resistance to most of the antibiotics tested. The highest resistance was seen against tetracycline (80.3%), while the isolates were the least resistant to nitrofurantoin (3.2%) (Table 1).

Differences were observed in the percentage resistance of each species tested in this study to antibiotic panel *E. faecalis* (79.6%), *E. casseliflavus* (85.7%), and other *Enterococcus* spp. (96.9%) were most resistant to tetracycline, while resistance in *E. faecium* was most observed against sulphamethoxazole-trimethoprim (84.2%) (Table 1).

Isolates resistant to one or more antibiotics in at least three different antibiotics classes were defined as multidrug-resistant (MDR). Among all the isolates, 222 (78%) were MDR, with a total of 47 antibiograms being identified (Table S1). Of the MDR isolates, 176 (79.3%) were *E. faecalis*, 13 (5.9%) *E. faecium*, 5 (2.3%) *E. casseliflavus*, 1 (0.5%) was *E. gallinarum*, and 27 (12.1%) were other *Enterococcus* spp. *E. faecalis* showed 38 antibiograms while *E. faecium*, *E. casseliflavus*, and *E. gallinarum* showed eight, four, and one antibiogram, respectively. The other *Enterococcus* spp. showed nine antibiograms (Table S1).

### 3.3. Prevalence of Antibiotic Resistance Genes

Phenotypic resistance to tetracycline, aminoglycosides, and macrolides was corroborated by the presence of the *tetM*, *aph(3′)-IIIa*, and *ermB* genes in 99.1%, 96.1%, and 88.3% of the isolates, respectively. *E. faecalis* harbored the highest number of resistance genes compared to the other species (Table 2). Tetracycline resistance was mostly associated with the presence of *tet*M (99.1%) than *tetK* (17.1%). Gentamicin resistance, associated with the gene *aac(6′)-Ie-aph(2″)-Ia*, was only detected in two *E. faecalis* isolates. The *aph(3′)-IIIa* gene was present in 96.1% of isolates with high percentage resistance to streptomycin and was mostly detected in *E. faecalis* isolates (76.0%) than in the other species.

### 3.4. Detection of Virulence of Factors

The most detected virulence gene among all isolates in the current study was *gelE* (89.1%), while the least detected gene was *efaAfm* (3.5%) (Table 3). No virulence genes were detected in *E. gallinarum*, while all the genes tested were identified in at least one *E. faecium* isolate.

### 3.5. Clonal Relatedness

**Figure 3:**
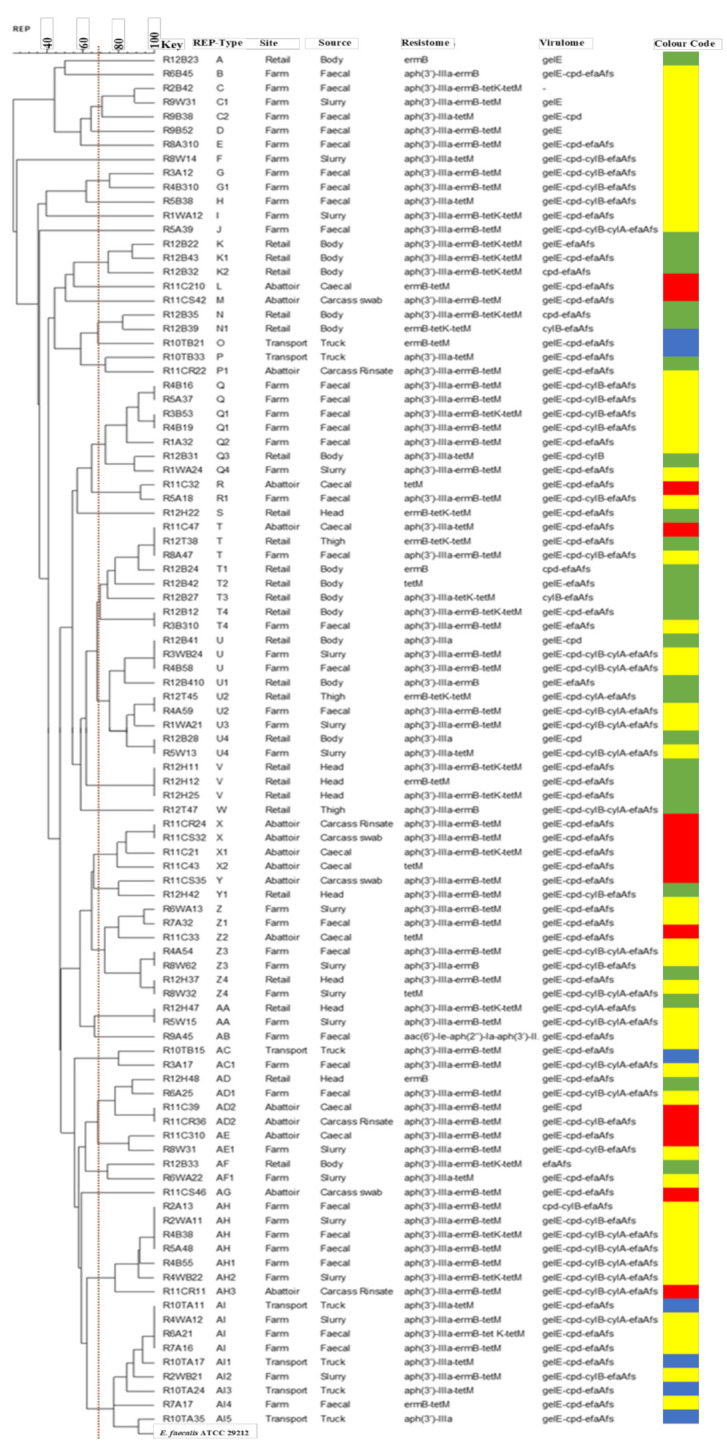
Dendrogram showing REP-types of *E. faecalis* isolates, based on ≥70% similarity index recovered along the farm-to-fork continuum. Farm = yellow, transport = blue, abattoir = red, retail = green. *E. faecalis* ATCC 29212 was used as the reference strain.

The evolutionary relationships of selected MDR *E. faecalis* isolates (*n* = 99) and all *E. faecium* isolates (*n* = 19) were determined using REP-PCR. The *E. faecalis* isolates were chosen based on their antibiograms and isolation source such that isolates with the same antibiogram from each sampling point along the farm-to-fork continuum were represented. *E. faecalis* displayed 35 REP-types (A-AI), the six major REP-types being U and AI (nine isolates each), T (eight isolates), Z, Q, and AH (seven isolates each). *E. faecalis* isolates from the “farm” were represented in all major REP-types (Figure 3). However, it must be noted that they were from different rounds of sampling on the farm (Round 1–Round 8). *E. faecalis* isolates from “transport” were also represented in major REP-type AI, while isolates from the “abattoir” and “retail” were quite diverse with representation in REP-types AH, T, Z, X and AD in the former and REP-types K, N, Q, T, U, V, and Z in the latter. (Figure 3).

**Figure 4:**
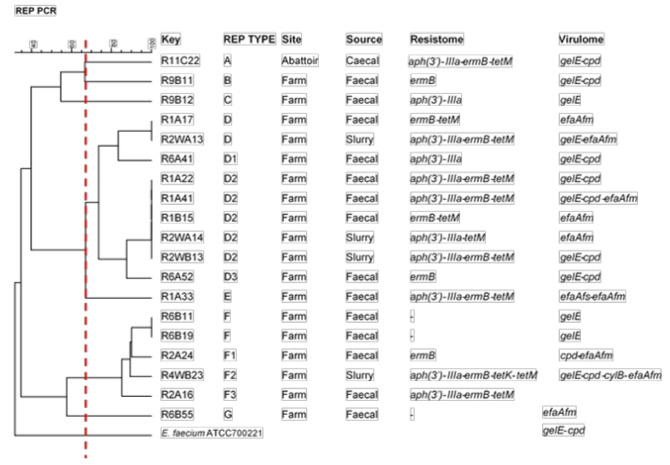
Dendrogram showing REP-types of *E. faecium* isolates, based on ≥70% similarity index recovered along the farm-to-fork continuum. E. faecium ATCC 700221 was used as the reference strain.

*E. faecium* was grouped into 7 REP-types (A-G), with two major REP-types consisting of 73.7% (*n* = 14) of *E. faecium* isolates, namely: D (nine isolates) and F (five isolates) (Figure 4). Of the 19 *E. faecium* isolates, 18 were from the farm, and one was from the abattoir. The largest clonal cluster showing a similarity index of 100% was D2 consisting of 5 isolates originating from fecal (Round 1) and wastewater (Round 2) samples. There was less diversity in the source of *E. faecium* isolates. Isolates belonging to the same REP-types were isolated from the farm and its environments (feces and wastewater).

## 4. Discussion

This study investigated the molecular epidemiology of *Enterococcus* spp. isolated from pigs in food production along the farm-to-fork continuum over four months in KwaZulu Natal, South Africa. A total of 284 isolates were obtained along the farm-to-fork continuum. Molecular screening confirmed 79.2% of the isolates as *E. faecalis*, *E. faecium*, *E. casseliflavus*, *E. gallinarum*, and other *Enterococcus* spp. The isolates displayed different antibiotic resistance percentages, with the highest resistance being to sulfamethoxazole-trimethoprim, while no resistance was recorded to vancomycin, teicoplanin, tigecycline, and linezolid. Also, 78% of the resistant isolates were MDR. Phenotypic resistance to tetracycline, aminoglycosides, and macrolides was corroborated by the presence of the *tet*M, *aph(3′)-IIIa*, and *erm*B genes in most of the isolates. Furthermore, most isolates harbored at least one of the virulence genes tested. Finally, the clonality revealed highly genetically diverse isolates along the continuum.

### 4.1. Isolation and Identification of Enterococcus spp.

*Enterococcus* species have been isolated from animals, including pigs, in numerous studies worldwide [18,19,20,21,22,23,24,25,26]. However, not all species have been reported at equal frequencies. In the current study, the prevalence of *E. faecalis* (79.2%) dominated across all sampling points, followed by *E. faecium* (6.7%), *E. casseliflavus* (2.5%), and *E. gallinarum* (0.4%). A considerable percentage (11.2%) was undifferentiated and classified as “other *Enterococcus* species” (Figure 2). The pattern of enterococcal species dominance recorded in the current study is similar to those reported in Malaysia [20] and Nigeria [25]. However, this observation differed from other studies’ reports, where *E. faecium* was more dominant [21,26]. For example, in a European study conducted by de Jong et al., the authors reported prevalences of 36% and 24% for *E. faecium* and *E. faecalis*, respectively [21]. Similarly, a previous South African study recorded *E. faecium* (37.5%) and *E. hirae* (31.25%) as the most dominant species in fecal samples collected from two pig farms [26]. The differences between the current studies and other studies could be due to the samples collected and the sampling approach used. The current study used a farm-to-fork approach, allowing samples to be collected from the farm through the transport system, the slaughter, to final packaging. On the other hand, the previous studies only collected samples from animals on the farm and farm environment. Furthermore, the current study only focused on pigs, while the other studies also included other animals such as cattle and chicken [21]. Nevertheless, the differences in the composition of the enterococcal populations could reflect differences in geographical regions, although it is difficult to draw such firm conclusions, highlighting the importance of including *Enterococcus* spp. in food animals’ surveillance programs.

The higher prevalence of *Enterococcus* species in the farm samples (feces and slurry) (Figure 1) was not surprising, as enterococci are normal inhabitants of the gut of humans and other animals [8,9]. However, the presence of enterococci in meat is undesirable [27], and at times, it can indicate the presence of salmonella in processed meat [27]. Thus, the isolation of these bacteria in the meat portions at the packaging stage, although at a lower prevalence, could represent a food safety issue as they could be transmitted to humans through the consumption of poorly cooked pork and other processed pork products. Therefore, appropriate measures must be implemented to eliminate all microbial contamination in the final products before dispatching them to the markets.

### 4.2. Antibiotic Susceptibility Profiles and Detection of Antibiotic Resistance Genes

The incidence of highly resistant enterococci has increased in recent years [15,23,26,28]. Intensive pig farming could facilitate the transmission of these resistant bacteria due to the proximity between the farmers, animals, and the environment in such settings [20]. In the current study, over 80% of the isolates were resistant to tetracycline (Table 1). Also, about 80% of all the resistant isolates were multidrug-resistant, displaying 47 different antibiograms (Table S1). Tetracycline and tylosin (one of four growth promoters banned in the European Union) are still approved as antibiotics for growth promotion under the Fertilizers, Farm Feeds, Agricultural Remedies and Stock Remedies Act (Act 36 of 1947) in South Africa. The majority of antibiotics consumed in food animals in South Africa include tetracycline, sulfonamides/trimethoprim, macrolides, penicillins, and cephalosporins which are of direct importance in human medicine [29]. This could explain the high percentage of resistance displayed by enterococcal isolates in this study to tetracycline, sulfamethoxazole-trimethoprim, erythromycin, and streptomycin. Using these antibiotics in the food animals could exert selection pressure for the development/escalation of resistance [15].

The most frequently encountered tetracycline resistance determinant in phenotypically tetracycline-resistant enterococci is *tet*M, even in clinical isolates [30], consistent with the current study’s findings. Almost all the isolates phenotypically resistant to tetracycline harbored the *tet*M gene (Table 2). Overall, 78 enterococcal isolates (34.7%) harbored both *tet*M (ribosomal protection) and *tet*K (efflux pump) resistance genes. The transferability of tetracycline resistance determinants has been associated with conjugative transposons, mainly Tn916/Tn1545 carrying the *tet*M gene, usually combined with *erm*B, although it has also been found on plasmids [11,31]. The *erm*B gene is the most reported erythromycin resistance determinant in enterococci [32]. More than 70% of all the isolates were phenotypically resistant to erythromycin in the current study (Figure 2). Of these, 88.3% harbored the *erm*B gene (Table 2). Furthermore, 181 enterococcal isolates co-carried the *tet*M and *erm*B genes. Other resistance genes like *erm*A, *erm*C, *erm*F, or *erm*T or the macrolide efflux pump could be also have been associated with erythromycin resistance observed in our study [33]. However, these were not examined in the current study.

Enterococci have intrinsic low-level resistance to aminoglycosides. Thus, combining cell wall-active agents like penicillin or glycopeptides with an aminoglycoside is commonly used to treat enterococcal infections [34]. However, enterococci with acquired aminoglycoside resistance challenge this treatment option by eliminating this synergistic effect. High level aminoglycoside resistance was detected in enterococcal isolates (streptomycin 62.6% (*n* = 204) and gentamicin 14% (*n* = 39). Two isolates from the high-level gentamicin resistance (HLGR) phenotype harbored the *aac(6′)-e-aph(2″)-Ia* gene. Other genes such as *aph(2′)-lc* and *aac(6′)-li* could have also contributed to the HLGR [33]. The *aph(3′)-IIIa* gene was detected in 96.1% of isolates with a high-level streptomycin resistance phenotype, suggesting a close association between phenotypic and genotypic resistance in our study isolates. Antibiotic resistance to quinupristin-dalfopristin was observed in 78.9% (*n* = 15) of *E. faecium* isolates. This is important as streptogramins such as quinupristin-dalfopristin are used to treat severe vancomycin-resistant *E. faecium* infections associated with bacteremia [35].

Despite the considerable percentage resistance recorded in the current study, all enterococcal isolates (100%) were susceptible to ampicillin, imipenem, teicoplanin, vancomycin, tigecycline, and linezolid. According to the WHO CIA List, these antibiotics are considered critically important in human health, among which vancomycin is classified as a “highest priority” antibiotic [36]. Susceptibility to several critically important antibiotics has also been reported in other studies [24,37]. The absence of resistance to “critically important” antibiotics for human medicines such as linezolid, imipenem, tigecycline, and vancomycin is encouraging.

### 4.3. Determination of Virulence Potentials of Enterococcus spp.

Enterococci, especially *E. faecalis* and *E. faecium*, are bacteria of a public health concern due to their increasing involvement in nosocomial infections [38]. This highlights the significance of their presence in food animals, as these could then serve as reservoirs and transmission routes for these pathogens. Also, identifying the virulence factors they harbor may help understand their complex pathogenic potentials [39]. In the current study, *E. faecalis* and *E. faecium* were positive for their respective cell wall adhesin genes, *efaAfs* and *efaAfm*. There was a high prevalence of gelatinase (*gelE*) and sex pheromone (*cpd*) genes, while cytolysin (*cylB* and c*ylA*) genes were detected to a lesser extent in these species (Table 3). These genes confer pathogenicity by coding for factors that degrade host tissue, favour biofilm formation (*gelE*), promote plasmid accumulation (*cpd*), and enhance haemolytic and bactericidal activity (*cylB* and *cylA*). Other studies have reported the *gelE* gene as the most prevalent virulence determinant among *E. faecalis* isolates from pigs in China and Korea [22,40]. Iweriebor et al. [26] also reported a high prevalence of *gelE* and *ace* genes from pigs in Eastern Cape, South Africa. It was also observed in the current study that most isolates carried more than two virulence determinants (87.7%). These findings suggest that the isolates reported in the current study could cause infection in susceptible human hosts upon exposure, and therefore their presence in intensive pig farming would require prompt attention.

### 4.4. Clonal Relatedness

REP-PCR was used to distinguish clonal relatedness among enterococcal isolates. MDR *E. faecalis* isolates along the farm-to-fork continuum were diverse with 35 REP-types (A-AI) than *E. faecium* isolates, with 7 REP-types (A-G). Regarding *E. faecalis*, it is interesting to note that REP-type T consisted of isolates from the farm that were ≥70% genetically related to those originating from the abattoir and retail meat. While this may suggest possible transmission of these isolates along the different stages of the production process, it must be noted that these isolates did not share the same resistance genes and virulence factors. REP-types Z, AH, AI comprised of fecal and wastewater isolates that were ≥70% genetically related, indicating possible enterococcal contamination of the associated environment. Studies have demonstrated that using untreated wastewater and animal feces on croplands could result in the environmental dissemination of resistance and virulence determinants via horizontal gene transfer, which could, in turn, disseminate resistance and virulence determinants back to animals or humans through crops [25]. This reinforces the necessity for a multisectoral approach for AMR surveillance programmes like the WHO’s One Health approach.

Furthermore, REP-type AI consisted of isolates from the farm that were ≥70% genetically related to those originating from the transport site. These isolates were obtained from truck swabs after the pigs were loaded onto the truck. This may indicate a possible transfer from the pigs to the truck; however, these isolates showed no clonal relation to isolates from further along the continuum (abattoir and retail), highlighting the importance of biosecurity measures, like in-house decontamination of the truck, would be necessary. However, further studies involving more resolute typing methods, such as whole-genome sequencing (WGS), will be needed to validate these claims. In comparison, *E. faecium* isolates showed a less diverse evolutionary relationship.

## 5. Conclusions

To the best of our knowledge, this is the first study in South Africa to investigate the molecular epidemiology of *Enterococcus* spp. isolated from pigs in food production along the farm-to-fork continuum in KwaZulu-Natal. The results of this study highlight the prevalence of enterococcal species, including MDR ones that harbored resistance and virulence genes in different permutations and combinations, suggesting that intensive pig farming can act as a reservoir for the potential transfer of these bacteria and their associated genes from pigs on the farm to occupationally exposed workers on the farm via direct contact with animals. Transmission could also extend human dining tables in households through animal products unless appropriate measures are taken to stop such transmission. The results highlight the importance of more robust guidelines for antibiotic use in intensive farming practices and the necessity of including *Enterococcus* spp. in food animal AMR surveillance programs. Further studies comparing antibiotic-fed and antibiotic-free pigs would shed more light on the possibility of reducing antibiotics use.

## Figures and Tables

**Table 1:** Susceptibility profiles of *Enterococcus* species to the antibiotics panel.

Antibiotics	*E. faecalis* (*n* = 225)			*E. faecium* (*n* = 19)			*E. casseliflavus* (*n* = 7)			*E. gallinarum* (*n* = 1)			Other *Enterococcus* spp. (*n* = 32)			Total (*n* = 284)		
	S	I	R	S	I	R	S	I	R	S	I	R	S	I	R	S	I	R
CIP	204 (90.7)	0 (0)	21 (9.3)	16 (84.2)	0 (0)	3 (15.8)	7 (100)	0 (0)	0 (0)	1 (100)	0 (0)	0 (0)	30 (93.8)	0 (0)	2 (6.3)	258 (90.8)	0 (0)	26 (9.2)
GEN	191 (84.9)	0 (0)	34 (15.1)	19 (100)	0 (0)	0 (0)	6 (85.7)	0 (0)	1 (14.3)	1 (100)	0 (0)	0 (0)	28 (87.5)	0 (0)	4 (12.5)	245 (86.3)	0 (0)	39 (13.7)
STR	68 (30.2)	0 (0)	157 (69.8)	5 (26.3)	0 (0)	14 (73.7)	2 (28.6)	0 (0)	5 (71.4)	0 (0)	0 (0)	1 (100)	5 (15.6)	0 (0)	27 (84.4)	80 (28.2)	0 (0)	204 (71.8)
Q-D *				4 (21.1)	0 (0)	15 (78.9)										4 (21.1)	0 (0)	15 (78.9)
NIT	218 (96.9)	0 (0)	7 (3.1)	17 (89.5)	0 (0)	2 (10.5)	7 (100)	0 (0)	0 (0)	1 (100)	0 (0)	0 (0)	32 (100)	0 (0)	0 (0)	275 (96.8)	0 (0)	9 (3.2)
SXT	50 (22.2)	0 (0)	175 (77.8)	3 (15.8)	0 (0)	16 (84.2)	2 (28.6)	0 (0)	5 (71.4)	1 (100)	0 (0)	0 (0)	13 (40.6)	0 (0)	19 (59.4)	69 (24.3)	0 (0)	215 (75.7)
ERY	8 (3.6)	56 (24.9)	161 (71.6)	0 (0)	6 (31.6)	13 (68.4)	0 (0)	3 (42.9)	4 (57.1)	0 (0)	0 (0)	1 (100)	1 (3.1)	5 (15.6)	26 (81.3)	9 (3.2)	70 (24.6)	205 (72.2)
TET	36 (16)	10 (4.4)	179 (79.6)	7 (36.8)	1 (5.3)	11 (57.9)	1 (14.3)	0 (0)	6 (85.7)	0 (0)	0 (0)	1 (100)	1 (3.1)	0 (0)	31 (96.9)	45 (15.8)	11 (3.9)	228 (80.3)
CHL	126 (56)	42 (18.7)	57 (25.3)	13 (68.4)	4 (21.1)	2 (10.5)	4 (57.1)	1 (14.3)	2 (28.6)	0 (0)	0 (0)	1 (100)	17 (53.1)	7 (21.9)	8 (25)	160 (56.3)	54 (19)	70 (24.6)
LEV	215 (95.6)	0 (0)	10 (4.4)	19 (100)	0 (0)	0 (0)	7 (100)	0 (0)	0 (0)	1 (100)	0 (0)	0 (0)	31 (96.9)	0 (0)	1 (3.1)	273 (96.1)	0 (0)	11 (3.9)

Percentages are presented in brackets. S = Susceptible, standard dosing regimen; I = Susceptible, increased exposure; R = Resistant * Q-D was only tested against *E. faecium*. Only antibiotics to which resistance was observed are reported. CIP = Ciprofloxacin, GEN = Gentamicin, STR = Streptomycin, TEC = Teicoplanin, Q-D = Quinupristin-Dalfopristin, NIT = Nitrofurantoin, SXT = Sulphamethoxazole-trimethoprim, ERY = Erythromycin, TET = Tetracycline, CHL = Chloramphenicol, LEV = Levofloxacin.

**Table 2:** Distribution of antibiotic resistance genes in phenotypically resistant *Enterococcus* spp.

*Enterococcus* spp.	*Antibiotic Resistance Genes*				
	*erm*B (*n* = 205)	*aph(3′)-IIIa* (*n* = 204)	*tet*K (*n* = 228)	*tet*M (*n* = 228)	*aac(6′)-Ie-aph(2″)-Ia* (*n* = 39)
*E. faecalis*	139 (67.8%)	155 (76.0%)	35 (15.4%)	177 (77.6%)	2 (5.1%)
*E. faecium*	13 (6.3%)	11 (5.4%)	1 (0.4%)	11 (4.8%)	0 (0.0%)
*E. casseliflavus*	2 (1.0%)	4 (2.0%)	0 (0.0%)	6 (2.6%)	0 (0.0%)
*E. gallinarum*	1 (0.5%)	0 (0.0%)	0 (0.0%)	1 (0.4%)	0 (0.0%)
*Other Enterococcus* spp.	26 (12.7%)	26 (12.7%)	3 (1.3%)	31 (13.6%)	0 (0.0%)
Total	181 (88.3%)	196 (96.1%)	39 (17.1%)	226 (99.1%)	2 (5.1%)

**Table 3:** Prevalence of virulence genes in all *Enterococcus* spp.

*Enterococcus* spp.	Virulence Genes					
	*efaAfs*	*gel*E	*cpd*	*cyl*B	*cyl*A	*efaAfm*
*E. faecalis* (*n* = 225)	201 (89.3%)	208 (92.4%)	186 (82.7%)	80 (35.6%)	49 (21.8%)	1 (0.4%)
*E. faecium* (*n* = 19)	0 (0.0%)	13 (68.4%)	10 (52.6%)	1 (5.3%)	0 (0.0%)	9 (47.4%)
*E. casseliflavus* (*n* = 7)	3 (42.9%)	5 (71.4%)	2 (28.6%)	0 (0.0%)	0 (0.0%)	0 (0.0%)
*E. gallinarum* (*n* = 1)	0 (0.0%)	0 (0.0%)	0 (0.0%)	0 (0.0%)	0 (0.0%)	0 (0.0%)
*Other Enterococcus* spp. (*n* = 32)	19 (59.4%)	27 (84.4%)	21 (65.6%)	8 (25.0%)	3 (9.4%)	0 (0.0%)
Total (*n* = 284)	223 (78.5%)	253 (89.1%)	219 (77.1%)	89 (31.3%)	52 (18.3%)	10 (3.5%)

## Data Availability

Data is contained within the article.

## Supplementary Materials

The following is available online at https://www.mdpi.com/article/10.3390/microorganisms9050882/s1, Table S1: Antibiogram displayed by multidrug-resistant *Enterococcus* spp.
