# Supplementary material for: From the Farms to the Dining Table: The Distribution and Molecular Characteristics of Antibiotic-Resistant Enterococcus spp. in Intensive Pig Farming in South Africa
Source: Microorganisms. 2021 Apr 21;9(5):882. doi: 10.3390/microorganisms9050882 (PMC8142977; doi:10.3390/microorganisms9050882)
Supplement: Supplementary file 1 [file microorganisms-09-00882-s001.zip › microorganisms-1148371-supplementary.pdf]

microorganisms

Supplementary materials

From the farms to the dining table: the distribution and molecular characteristics of antibiotic-resistant *Enterococcus* spp. in intensive pig farming in South Africa

Sasha Badul [1], Akebe Luther King Abia [1], Daniel G. Amoako [1,2,3], Keith Perrett [4], Linda A. Bester [2] and Sabiha Y. Essack [1*]

[1] Antimicrobial Research Unit, College of Health Sciences, University of KwaZulu-Natal, Durban 4000, South Africa; sashabadul@yahoo.co.uk (S.B.); lutherkinga@yahoo.fr (A.L.K.A.); amoakodg@gmail.com (D.G.A.); essacks@ukzn.ac.za (S.Y.E.).
[2] Biomedical Resource Unit, College of Health Sciences, University of KwaZulu-Natal, Durban 4000, South Africa; besterl@ukzn.ac.za (L.A.B.).
[3] Centre for Respiratory Diseases and Meningitis, National Institute for Communicable Diseases, Johannesburg 2131, South Africa.
[4] Epidemiology Section, KwaZulu-Natal Agriculture & Rural Development-Veterinary Service; Pietermaritzburg 3201, South Africa; keith.perrett@kzndard.gov.za (K.P.).
* Correspondence: essacks@ukzn.ac.za

Table S1. Antibiograms displayed by multidrug-resistant *Enterococcus* spp.

Antibiogram	*E. faecalis* (*n* = 225)	*E. faecium* (*n* = 19)	*E. casseliflavus* (*n* = 7)	*E. gallinarum* (*n* = 1)	*Enterococcus spp.* (*n* = 32)
CIP-GEN-STR-SXT-ERY-TET	1	0	0	0	0
CIP-GEN-STR-SXT-ERY-TET-CHL	1	0	0	0	1
CIP-STR-ERY-TET	3	0	0	0	0
CIP-STR-ERY-TET-CHL	1	0	0	0	0
CIP-STR-NIT-ERY-TET	1	0	0	0	0
CIP-STR-NIT-SXT-ERY-TET	1	0	0	0	0
CIP-STR-Q-D-SXT-ERY-TET	0	1	0	0	0
CIP-STR-SXT-ERY-TET	2	0	0	0	0
CIP-STR-SXT-ERY-TET-CHL-LEV	2	0	0	0	0
CIP-STR-SXT-ERY-TET-LEV	2	0	0	0	0
CIP-SXT-TET	1	0	0	0	0
CIP-SXT-TET-CHL-LEV	1	0	0	0	0
CIP-SXT-TET-LEV	2	0	0	0	1
ERY-TET-CHL	2	0	0	0	0
GEN-STR-CHL	1	0	0	0	0
GEN-STR-ERY-TET	0	0	0	0	1
GEN-STR-NIT-SXT-ERY-TET	1	0	0	0	0
GEN-STR-SXT-CHL	1	0	0	0	0
GEN-STR-SXT-ERY-TET	27	0	0	0	2
GEN-STR-SXT-ERY-TET-CHL	2	0	1	0	0
NIT-ERY-SXT	1	0	0	0	0
NIT-SXT-ERY-TET	1	0	0	0	0
NIT-SXT-TET	1	0	0	0	0

Q-D-SXT-ERY-CHL	0	1	0	0	0
STR-ERY-CHL	1	0	0	0	0
STR-ERY-TET	16	0	1	0	7
STR-ERY-TET-CHL	7	0	0	1	2
STR-NIT-ERY-TET-CHL	1	0	0	0	0
STR-NIT-SXT-ERY-TET	2	0	0	0	0
STR-NIT-SXT-ERY-TET-CHL	1	0	0	0	0
STR-Q-D-ERY	0	1	0	0	0
STR-Q-D-ERY-TET	0	1	0	0	0
STR-Q-D-NIT-SXT-ERY-TET	0	1	0	0	0
STR-Q-D-NIT-SXT-ERY-TET-CHL	0	1	0	0	0
STR-Q-D-SXT-ERY-TET	0	6	0	0	0
STR-Q-D-SXT-TET	0	1	0	0	0
STR-SXT-CHL	1	0	0	0	0
STR-SXT-ERY	5	0	0	0	0
STR-SXT-ERY-TET	31	0	2	0	8
STR-SXT-ERY-TET-CHL	24	0	0	0	4
STR-SXT-ERY-TET-CHL-LEV	2	0	0	0	0
STR-SXT-TET	11	0	1	0	0
STR-SXT-TET-CHL	5	0	0	0	0
SXT-ERY-CHL	4	0	0	0	0
SXT-ERY-TET	8	0	0	0	0
SXT-ERY-TET-CHL	1	0	0	0	1
SXT-TET-CHL	1	0	0	0	0
Total (*n* = 222; 78%)	176 (78%)	13 (68%)	5 (71%)	1 (100%)	27 (84%)

*Q-D was only tested against E. faecium. Only antibiotics to which resistance was observed are reported. CIP = Ciprofloxacin, GEN = Gentamicin, STR = Streptomycin, TEC = Teicoplanin, Q-D = Quinupristin-Dalfopristin, NIT = Nitrofurantoin, SXT = Sulphamethoxazole-trimethoprim, ERY = Erythromycin, TET = Tetracycline, CHL = Chloramphenicol, LEV = Levofloxacin.
